# Correction: Neurologic manifestations in hospitalized patients with COVID-19 in Mexico City

**DOI:** 10.1371/journal.pone.0315306

**Published:** 2024-12-05

**Authors:** Fernando Daniel Flores-Silva, Miguel García-Grimshaw, Sergio Iván Valdés-Ferrer, Alma Poema Vigueras-Hernández, Rogelio Domínguez-Moreno, Dioselina Panamá Tristán-Samaniego, Anaclara Michel-Chávez, Alejandra González-Duarte, Felipe A. Vega-Boada, Isael Reyes-Melo, Amado Jiménez-Ruiz, Oswaldo Alan Chávez-Martínez, Daniel Rebolledo-García, Osvaldo Alexis Marché-Fernández, Samantha Sánchez-Torres, Guillermo García-Ramos, Carlos Cantú-Brito, Erwin Chiquete

An additional affiliation is missing for the first author. Fernando Daniel Flores-Silva is also affiliated with the Programa de Maestría y Doctorado en Ciencias Médicas, Odontológicas y de la Salud, Universidad Nacional Autónoma de México, Mexico City, Mexico.
