# Supplementary material for: Neurologic manifestations in hospitalized patients with COVID-19 in Mexico City
Source: PLoS One. 2021 Apr 8;16(4):e0247433. doi: 10.1371/journal.pone.0247433 (PMC8031187; doi:10.1371/journal.pone.0247433)
Supplement: Attachment — Submitted filename: Response to reviewers.doc [file pone.0247433.s002.doc]

**AUTHOR’S REVISION LETTER**

***Revision 1***

**Neurological manifestations in hospitalized patients with COVID-19 in Mexico City**

# *Fernando Daniel Flores-Silva, Miguel García-Grimshaw, Sergio Iván Valdés-Ferrer, Alma Poema Vigueras-Hernández, Rogelio Domínguez-Moreno, Dioselina Panamá Tristán-Samaniego, Anaclara Michel-Chávez, Alejandra González-Duarte, Felipe Vega-Boada, Isael Reyes-Melo, Amado Jiménez-Ruiz, Oswaldo Alan Chávez-Martínez, Daniel Rebolledo-García, Osvaldo Alexis Marché-Fernández, Samantha Sánchez-Torres, Guillermo García-Ramos, Carlos Cantú-Brito, Erwin Chiquete*

# We have analyzed the reviewers’ suggestions to our previous paper. We completely agree with these observations and have performed the changes in our new manuscript accordingly. Changes that apply in this revised version are highlighted.

# We profoundly appreciate the kind attention that the reviewers and the editorial team have given to our manuscript.

**Response to reviewers**

**Editorial team comments**

a) If there are ethical or legal restrictions on sharing a de-identified data set, please explain them in detail (e.g., data contain potentially identifying or sensitive patient information) and who has imposed them (e.g., an ethics committee). Please also provide contact information for a data access committee, ethics committee, or other institutional body to which data requests may be sent.

*Response*: We have consulted the Ethics Committee and as long as personal identification data are removed, there is no problem with data sharing.

b) If there are no restrictions, please upload the minimal anonymized data set necessary to replicate your study findings as either Supporting Information files or to a stable, public repository and provide us with the relevant URLs, DOIs, or accession numbers. Please see http://www.bmj.com/content/340/bmj.c181.long for guidelines on how to de-identify and prepare clinical data for publication. For a list of acceptable repositories, please see http://journals.plos.org/plosone/s/data-availability#loc-recommended-repositories.

*Response*: We will submit the proper database in the corresponding field.

**Reviewer 1**

Overall, a very nice & timely cross-sectional study that provides important insight in to the neurologic manifestations of the COVID-19. The manuscript is well written and precise. It certainly deserves publication.

I see no need for major revisions.

I have 2 suggestions for minor revisions: (1) I would recommend a more explicit statement of the study design be included in the methods section. That the study is a cross-sectional analysis is mentioned in the introduction but should be explicitly stated in the methods. (2) Although the comparison groups are evident from the Tables, it is not clear from the text of the manuscript. The addition of text in the methodology ("The study aims to describe differences in demographics, clinical presentation, and disease severity in those patients who experienced neurologic events and those who did not. For these purposes, we define patients with neurologic events as those who suffered delirium, delayed recovery...") or something similar. This addition will lend more clarity to the text.

*Response (1)*: We agree with this suggestion and have made the changes accordingly.

*Response (2)*: We completely agree. This amendment was already made in the new version of the paper

Also, note that on p.7 of the submission, "Data Collection" section, there is a missing comma between pulmonary and neurological. Neurologic (rather than neurological - meaning pertaining to the study of or discipline of neurology) would be the preferred usage.

*Response*: Thank you very much. We have corrected these errors in our revised paper.

**Reviewer 2**

The authors have done a nice job in describing the neurological manifestations of COVID19 in Mexico City. I have no critiques or suggestions. I'm not sure what the minimum character count is, but i'm pasting in the abstract to fulfill it.

*Response*: Thank you. We will check for the words/character count of the Abstract in detail and will make the amendments accordingly.
